# Eating Patterns and Dietary Interventions in ADHD: A Narrative Review

**DOI:** 10.3390/nu14204332

**Published:** 2022-10-16

**Authors:** Sofia Pinto, Teresa Correia-de-Sá, Benedita Sampaio-Maia, Carla Vasconcelos, Pedro Moreira, Joana Ferreira-Gomes

**Affiliations:** 1 Faculty of Nutrition and Food Sciences, University of Porto, 4150-180 Porto, Portugal; 2 Department of Biomedicine, Faculty of Medicine, University of Porto, 4200-319 Porto, Portugal; 3 INEB—Institute of Biomedical Engineering, University of Porto, 4200-135 Porto, Portugal; 4 i3S—Institute for Research & Innovation in Health, University of Porto, 4200-135 Porto, Portugal; 5 Faculty of Dental Medicine, University of Porto, 4200-393 Porto, Portugal; 6 Nutrition Service, University Hospital Center of São João, 4200-319 Porto, Portugal; 7 EPIUnit, Institute of Public Health, University of Porto, 4200-450 Porto, Portugal; 8 IBMC—Institute for Molecular and Cell Biology, University of Porto, 4200-135 Porto, Portugal

**Keywords:** ADHD, dietary interventions, dietary patterns

## Abstract

Attention Deficit Hyperactivity Disorder (ADHD) is one of the most common neurodevelopmental disorders in childhood, affecting ~7% of children and adolescents. Given its adverse health outcomes and high healthcare and societal costs, other treatment options beyond pharmacotherapy have been explored. Case-control studies have shown that dietary patterns may influence the risk of ADHD, and specific dietary interventions have been proposed as coadjuvant treatments in this disorder. These include nutritional supplements, gut microbiome-targeted interventions with biotics, and elimination diets. The purpose of this review is to examine which dietary patterns are most associated with ADHD and to summarize the existing evidence for the clinical use of dietary interventions. The literature showed that non-healthy dietary patterns were positively associated with ADHD, whereas healthy patterns were negatively associated. As for nutritional supplements, only vitamin D and vitamin D + magnesium appeared to improve ADHD symptoms when baseline levels of vitamin D were insufficient/deficient. Regarding biotics, evidence was only found for *Lactobacillus rhamnosus GG* and for multi-species probiotic supplementation. Elimination diets have scarce evidence and lead to nutritional deficiencies, so caution is advised. Overall, more robust scientific evidence is required for these dietary interventions to be implemented as part of ADHD therapy.

## 1. Introduction

Attention Deficit Hyperactivity Disorder (ADHD) is a neurodevelopmental disorder mainly characterized by hyperactivity, inattention, and impulsivity symptoms [1,2]. Beyond its adverse health outcomes [1], the healthcare and societal costs of the management of children and adolescents with ADHD makes it inevitable to search for other treatment options. In the United Kingdom, the mean cost per adolescent for the National Health Service, social care, and education resources in a 12-month period related to ADHD was GBP 5493 [3]. In Spain, the estimated average cost of ADHD, per year, per child/adolescent, was EUR 5733 in 2012, and pharmacotherapy accounted for 25.8% of direct costs and 15.5% of total costs [4].

The etiology of ADHD relies on both genetic and environmental factors [1]. Diet, as a modifiable environmental factor, has been investigated as a potential therapy option in ADHD. Studies have shown that children with ADHD show less adherence to healthy eating patterns than children without this disorder [5,6,7]. Moreover, dietary patterns may influence the risk of ADHD, since patterns described as “Junk-food”, “Processed”, “Snack”, “Sweet”, and “Western-like” have been positively associated with this pathology [5,6,8]. On the other hand, healthy eating patterns, such as the Mediterranean diet, Dietary Approaches to Stop Hypertension (DASH), and vegetarian diets, filled with vegetables and fruits and rich in micronutrients, have been inversely associated with the risk of ADHD [7,9]. Furthermore, since diet plays an important role in children’s health and development, school food environment policies may improve targeted dietary behaviors and therefore be a critical tool to promote healthy diets in children [10].

Recently, specific nutrients, such as vitamin D, zinc, iron, and polyunsaturated fatty acids (PUFAs), have been proposed as coadjuvants in the treatment of ADHD [11]. Numerous diet interventions such as elimination diets and dietary supplementation have also been investigated, but the results remain controversial since there is a lack of high-quality RCTs that corroborate the efficacy of these interventions [12,13].

The main objective of this review is to summarize the existing evidence for the clinical use of dietary interventions in ADHD patients, as well as reviewing which dietary patterns are most associated with ADHD.

## 2. Attention Deficit Hyperactivity Disorder (ADHD)

Attention Deficit Hyperactivity Disorder (ADHD) is one of the most common neurodevelopmental disorders in childhood, affecting about 7% of children and adolescents worldwide [14,15]. It begins in early childhood and, in most cases, persists into adulthood. Symptoms include hyperactivity, inattention, impulsivity, impaired executive function, and emotional dysregulation, with several comorbidities emerging along the developmental trajectory [1]. Although ADHD is highly heritable, studies suggest that its etiology is multifactorial, with both genetic and environmental factors reflecting its phenotypic heterogeneity [1].

Diagnosis of ADHD relies mostly on diagnostic criteria provided by the *Diagnostic and Statistical Manual of Mental Disorders*, 5th edition (DSM-5). According to the DSM-5, at least six symptoms of inattention, hyperactivity, or both, before the age of 12 years and in two or more settings, are required for the diagnosis of ADHD [2]. The symptoms exhibited must interfere with one’s functioning and may be categorized as predominantly inattentive, predominantly hyperactive, or combined. This assessment is based on a comprehensive clinical and psychosocial evaluation, as well as a full developmental and psychiatric history [16]. Rating scales, though not diagnostic, should also be used to collect supplementary information concerning the symptomatology and its severity in all settings and should be completed by different informants, namely the parents/caregivers, teachers, and/or other relevant adult figures [16,17]. Examples of validated rating scales widely used are the Conners Comprehensive Behavior Rating Scales, the NICHQ Vanderbilt Assessment Scale, the ADHD-RS-V, the SNAP-IV-26, and the Strengths and Difficulties Questionnaire (SDQ) [16,17].

Treatment of ADHD currently rests on pharmacotherapeutic interventions with the use of stimulant (methylphenidate and amphetamine formulations) and non-stimulant medications (selective alpha-2 adrenergic agonists: guanfacine, clonidine; and selective norepinephrine reuptake inhibitor: atomoxetine) [18,19], both of which have proven to effectively reduce ADHD symptomatology in children and adolescents [19]. These pharmacological approaches, however, have known side effects. Stimulants are associated with short-term adverse effects, such as decreased appetite, weight loss, insomnia, abdominal pain, headaches, and anxiety [17,19]. They also appear to cause a decrease in growth rate, especially for those on higher and more consistent doses, with no indication of a growth rebound [20]. As for non-stimulants, atomoxetine is associated with adverse effects, such as headaches, abdominal pain, decreased appetite, and somnolence in children, and adverse effects such as nausea, dry mouth, decreased appetite, and insomnia in adults [21]. Atomoxetine also seems to cause suicidal thoughts in some children [22], though an association between atomoxetine and increased risk of suicidality has yet to have been found [21,23,24]. Liver injuries, though extremely rare, have been connected to treatments with atomoxetine [24,25]. A link to growth delays has also been found, though it appears to be reversible over time [19,24,26]. For guanfacine, adverse effects include sedation, somnolence, fatigue, drowsiness, headaches, and upper abdominal pain [27]. Similarly, for clonidine, adverse effects include fatigue, irritability, pharyngolaryngeal pain, somnolence, headaches, and upper abdominal pain [28]. Both types of drugs (stimulant and non-stimulant) have effects on heart rate and blood pressure, but the risk of major adverse cardiovascular events is significantly low [17,19]. Psychosis and mania, though infrequent, have also been reported to occur in those treated with these types of medication [29].

Besides the aforementioned pharmacological treatments, non-pharmacological interventions, such as psychological therapies and diet, have also been used for the management of ADHD. The psychological therapies include behavioral therapy, cognitive training, and neurofeedback. Of these three, however, only behavioral therapy has shown statistically significant benefits and can be recommended as an evidence-based intervention [30,31]. In fact, behavioral therapy combined with stimulants appears to be more effective than treatment with stimulants or non-stimulants alone [30]. Regarding dietary interventions, they mostly focus on dietary supplements with vitamins, minerals, and PUFAs, microbiome-targeted interventions with pre-, pro-, and synbiotic supplementation, and restriction or elimination diets. More recently, studies have focused on dietary patterns with a more holistic approach, as treatment options for ADHD [6] and the most promising dietetic approaches in ADHD are, in fact, food patterns considered to be healthy (i.e., Mediterranean diet and DASH) and the Few-Foods Diet for children [13]. Still, the quality of evidence for the impact of non-pharmacological treatments in ADHD is moderately low for the time being, which highlights the need for future high-quality randomized trials [30,31,32].

## 3. Dietary Patterns

Dietary pattern analysis provides a more comprehensive understanding of the diet and nutrient interactions than looking at an isolated food or nutrient [33]. Changes in society have led to a global nutritional transition that affects dietary and eating patterns, with families having less time for food preparation and for eating together [34]. Diets began to shift towards increased reliance upon processed foods, increased take-away foods, edible oils, and sugar-sweetened beverages, and all these changes have had negative effects on the health of many populations around the world [34].

The relationship between dietary patterns and ADHD has yielded inconclusive results, with some studies showing that a healthy eating pattern could significantly decrease the risk of ADHD [35] and others showing no significant effect [36]. A recent systematic review and meta-analysis showed that, in fact, the type of diet ingested influences the risk of ADHD [6]. The authors demonstrated that a healthy dietary pattern with consumption of fruits and vegetables, fish, and high in PUFAs and micronutrients such as magnesium, zinc, and phytochemicals seems to decrease the risk of ADHD by 37% (OR: 0.63; 95% CI: 0.41–0.96). On the other hand, both the Western-type and the junk food dietary patterns, very characteristically consumed by children, increase the risk of ADHD. The Western pattern, rich in red and processed meats, refined cereal grains, soft drinks, and hydrogenated fats, was shown to increase the risk of ADHD by 92% (OR: 1.92; 95% CI: 1.13–3.26; *p*: 0.016) while the junk food pattern, characterized by a high consumption of processed foods, with high amounts of artificial food coloring (AFC) and sugar, was found to increase the risk by 51% (OR: 1.51; 95% CI: 1.06–2.16; *p*: 0.024). Indeed, the daily consumption of AFC has quadrupled in the last 50 years [37], and studies have shown they can affect the brain without crossing the blood–brain barrier [37,38]. In a double-blind, placebo-controlled crossover challenge with 225 mg AFC disguised in chocolate cookies or placebo chocolate cookies for 3 days each week, with testing on the third day each week, the authors found that AFC exposure may affect brainwave activity and ADHD symptoms in college students with ADHD [38]. However, a 2004 systematic review and meta-analysis showed that the overall effect size of AFCs on hyperactivity is 0.283 (95% CI, 0.079–0.488), falling to 0.210 (95% CI, 0.007–0.414) when the smallest and lowest quality trials were excluded [39].

Another systematic review [5] distinguished only two dietary patterns (healthy and unhealthy), and found that the healthy pattern had a protective effect (OR: 0.65; 95% CI: 044–0.97), while the unhealthy pattern increased the risk of ADHD by 41% (OR: 1.41; 95% CI: 1.15–1.74), even after stratifying studies by design (cohort, case-control, or cross-sectional), geographic area (Europe or Asia/Oceania), and sample size (*n* ≥ 1000 or *n* < 1000). On the other hand, the authors found that children with ADHD show less adherence to healthy eating patterns than children without this disorder. Ríos-Hernández et al. [7], in a case-control study with a total of 120 children (60 newly diagnosed with ADHD and 60 controls), also found that lower adherence to a Mediterranean diet was associated with ADHD diagnosis (OR: 7.07; 95% CI: 2.65–18.84) in a Spanish population. Although these cross-sectional associations do not establish causality, the authors raise the question of whether low adherence to a Mediterranean diet might play a role in ADHD development.

In Ma’anshan city in China, Yan et al. [40] did a cross-sectional survey, in a large sample of 14,912 children aged 3–6 years old, and assessed their usual dietary intake through a semi-quantitative food frequency questionnaire and ADHD symptoms based on the 10-item Chinese version of the Conners Abbreviated Symptom Questionnaire. The authors found five different dietary patterns and concluded that the “Processed” (OR = 1.56, 95% CI: 1.31–1.86) and “Snack” (OR = 1.76, 95% CI: 1.49–2.07) dietary patterns were significantly and positively associated with ADHD symptoms, while the “Vegetarian” (OR = 0.67, 95% CI: 0.56–0.79) pattern was negatively correlated with ADHD symptoms.

In conclusion, dietary patterns seem to play a potential role in the risk of ADHD, as patterns described as “Junk-food”, “Processed”, “Snack”, “Sweet”, and “Western-like” are the ones most positively associated with this pathology [5,6,8], whereas healthy eating patterns, such as the Mediterranean diet, are inversely associated with ADHD [7,9]. These data support the idea that not only specific nutrients or chemical food compounds but the whole diet should be considered in ADHD.

### Foods, Food Groups, and Nutrients

Within the dietary patterns, at the food level, both Western and junk food patterns contain high amounts of refined grains, processed foods, and sugar, which have been related to ADHD [8,41]. In a Korean study with 986 school-age children with ADHD and learning disabilities, the authors found that a high intake of sweetened desserts, fried foods, and salt was positively associated with learning, attention, and behavioral problems, whereas a balanced diet, with regular meals, and a high intake of dairy products and vegetables, was negatively associated with these problems [42].

In fact, Farsad-Naeimi et al. [43] found that higher consumption of sweetened beverages was associated with 40% greater odds (pooled effect size: 1.22, 95% CI: 1.04–1.42) of ADHD symptoms in children over 7 years old, compared with their lower intake counterparts. However, the authors also found that dietary sugars alone did not increase the risk of developing ADHD symptoms [43]. This association between sugar intake and ADHD remains conflicting in the literature, with some studies finding positive associations and others finding no significant associations [41,43,44].

Salvat et al. [45], in their sample of 100 children with ADHD, found that they ingested less protein, in percentage terms, but a greater amount of simple sugars (*p* = 0.007), tea (*p* = 0.006), and “ready-to-eat” meals (*p* = 0.002) than their control peers. The authors also showed a lower intake of vitamins B1, B2, and C, as well as zinc and calcium, by children with ADHD [45]. In fact, several studies have shown that children with ADHD have reduced plasma levels of trace elements such as zinc, copper, iron, magnesium, and selenium [46,47,48,49,50], which are essential for brain development and functioning [51,52,53]. Iron is an essential cofactor required for several functions, such as neurotransmitter metabolism, particularly dopamine production, which is a core factor in ADHD. Zinc is also an essential trace element, required for cellular functions related to the metabolism of neurotransmitters, melatonin, and prostaglandins. Altered levels of iron and zinc have been related with the aggravation and progression of ADHD [54].

Vitamins A and D have recently emerged as micronutrients relevant to ADHD, since serum concentrations of 25(OH)D and retinol were linked with this disorder after adjustment for age, Body Mass Index (BMI), season of blood sampling, and sun exposure, and this co-deficiency was associated with symptom severity [55]. A meta-analysis of five case-control studies found that lower vitamin D status is significantly associated with the likelihood of ADHD (OR: 2.57; 95% CI: 1.09–6.04), and the meta-analysis of prospective studies conducted in 4137 participants indicated that perinatal suboptimal vitamin D concentrations are significantly associated with a higher risk of ADHD in later life (RR: 1.40; 95% CI: 1.09–1.81) [56].

In addition, PUFAs are crucial for optimal neurotransmitter function, and some studies have confirmed that children and adolescents with ADHD have lower levels of eicosapentaenoic acid (EPA-C20:5), docosahexaenoic acid (DHA-C22:6), and total PUFA series *n*-3 in their blood and buccal tissues [57].

In summary, when looking into a food-based level, sweetened desserts, fried foods, and salt seem to be positively associated with ADHD [42]. Although the association between sugar intake and ADHD remains conflicting in the literature [43], it seems reasonable to limit its intake within a healthy eating pattern. Even taking a more microscopic look at a nutrient-based level, children with ADHD have reduced plasma levels of important brain function trace elements, such as zinc, copper, iron, magnesium, and selenium [46,47,48,49,50]. In addition, vitamins A and D and PUFAs have emerged as nutrients to take into consideration in this disorder [55,57].

## 4. Nutritional and Dietary Interventions

Regarding diet interventions as treatment options for ADHD, they mostly focus on dietary supplements with vitamins, minerals, and PUFAs, microbiome-targeted interventions with pre-, pro-, and synbiotics, and specific diets such as restriction or elimination diets.

### 4.1. Nutritional Supplements

A systematic review and meta-analysis of four randomized controlled trials (RCT), with a total of 256 children, addressing vitamin D supplementation as an adjunctive therapy to methylphenidate, demonstrated a small but statistically significant improvement in ADHD total scores, inattention, hyperactivity, and behavior scores, but no statistically significant improvement in oppositional scores [58]. Of all these RCTs, it is important to note that only Dehbokri et al. [59] assessed the baseline levels of vitamin D and found that children with a sufficient level at baseline did not show improvements in ADHD scores following 50,000 IU/week vitamin D supplementation for 6 weeks. This result could indicate that only ADHD children with insufficient or deficient vitamin D levels might benefit from this diet intervention and that monitoring their plasmatic levels of vitamin D should be a regular procedure. In summary, the findings of this meta-analysis conclude that there is clinical evidence that vitamin D supplementation may improve ADHD symptoms without any obvious side effects. The included RCTs in the meta-analysis were within safe and acceptable ranges of doses and duration of supplementation, since it has been reported that severe adverse effects could occur after 12 to 52 weeks supplementation of 40,000 IU/day [58].

Hemamy et al. [60] performed a randomized, double-blind, placebo-controlled clinical trial with 66 children with ADHD to test vitamin D and magnesium. The authors assessed children’s mental health at baseline and at the end of the study using the Strengths and Difficulties Questionnaire (SDQ) and found that co-supplementation for a duration of 8 weeks with 50,000 IU/week of 25-hydroxy-vitamin D3 and 6 mg/kg/day of magnesium significantly reduced emotional problems (*p* = 0.001), conduct problems (*p* = 0.002), peer problems (*p* = 0.001), the prosocial score (*p* = 0.007), total difficulties (*p* = 0.001), the externalizing score (*p* = 0.001), and the internalizing score (*p* = 0.001), compared with the placebo group. It is important to note that both groups were on methylphenidate (31.33 ± 9.93 mg/kg in the intervention group versus 31.21 ± 8.81 mg/kg in the control group); however, the results were adjusted for the effect of this therapy. This study showed that supplementing with vitamin D and magnesium could improve the behavioral function and mental health of medicated children with ADHD.

To the best of our knowledge, there is no evidence on the effect of dietary supplements with retinol in ADHD symptoms, but recently there has been a registered protocol for a randomized, double-blinded, placebo-controlled, multicentric trial in China [61] which aims to determine the effect of vitamin A and D supplementation, as an adjunctive therapy to methylphenidate, on ADHD symptoms. The authors have a target of 504 patients who will be followed for 8 weeks and will be allocated into three groups (vitamin AD, vitamin D, and placebo). This will be the first clinical trial to examine the effects of vitamin A and vitamin D co-supplementation in ADHD; however, there are still no published results of this RCT.

Dietary supplementation with zinc and iron was assessed in a systematic review of nine randomized clinical trials [54]. This systematic review showed that, compared with placebo, dietary supplementation with zinc and iron for 6 to 10 weeks was associated with improvements in ADHD severity at the end of the treatments. Although the effect size of the outcomes tended to be low and/or focused on specific ADHD symptoms/measures, they appear to be most consistent for zinc [54]. The role of zinc and iron as dopamine reuptake inhibitors, which is the same core target as the stimulant medications used in the combined treatment plans for the disorder, could potentially explain the path between zinc–iron levels and the various ADHD manifestations [54]. However, another systematic review [62] revealed, in 2013, that, when adjusting for baseline zinc levels, supplementation with zinc, either alone or in combination with stimulants, did not improve ADHD.

Table 1 summarizes the results of intervention trials with nutritional supplements (zinc, iron, and vitamin D) in ADHD.

### 4.2. PUFA Supplementation

Chang et al. [57], in their 2018 systematic review and meta-analysis, showed that *n*-3 PUFA supplementation improves total ADHD symptoms compared with placebo, but with a modest effect size (g = 0.38). However, the authors highlighted that only studies with EPA doses of >500 mg improve hyperactivity symptoms. *n*-3 PUFA supplementation showed efficacy in improving omission and commission errors, but not memory and information processing, in children with ADHD. On the other hand, Händel et al. [74], in their 2021 systematic review and meta-analysis, with a total of 31 relevant RCTs including 1755 patients, found that PUFA supplementation showed no effect on ADHD core symptoms rated by parents (k = 23; SMD: −0.17; 95% CI: −0.32, −0.02) or teachers (k = 10; SMD: −0.06; 95% CI: −0.31, 0.19). There was no effect on behavioral difficulties rated by parents (k = 7; SMD: −0.02; 95% CI: −0.17, 0.14) or teachers (k = 5; SMD: −0.04; 95% CI: −0.35, 0.26), and there was no effect on quality of life (SMD: 0.01; 95% CI: −0.29, 0.31). The populations in the included studies consisted of children with ADHD in the age group ranging from 6–18 years. The interventions consisted of supplements with PUFAs with either omega 3, omega 6, or combined with both types of fatty acids. The interventions lasted between 8 weeks to 12 months. In three of the included studies, children were also in medical treatment in both the intervention group and the placebo group, whereby fatty acid treatment was investigated as an active add-on treatment in the intervention group. The authors concluded that, for now, there seems to be no benefit of PUFA supplementation in ADHD treatment. Table 2 summarizes the results of the previously described meta-analyses of intervention trials with PUFAs in ADHD.

Still, in a 2021 randomized placebo-controlled trial evaluating the efficacy of an omega-3/omega-6 fatty acid supplement in 40 preschool children at risk for ADHD, Döpfner et al. [75] found more promising results. In this study, participants were treated with either two capsules of an omega-3/omega-6 fatty acid supplement twice a day, corresponding to a daily dose of 372 mg EPA, 116 mg DHA, and 40 mg gamma-linolenic acid (GLA), or a placebo for 4 months. Results following intention-to-treat (ITT) analyses suggested moderate effects of omega-3/omega-6 PUFAs on both parent- and teacher-rated overall ADHD symptoms, on teacher-rated inattention symptoms, and on parent-rated hyperactivity/impulsivity. The results of the analyses including all available data, however, did not suggest an effect on parent- or teacher-rated overall ADHD symptoms, but it did find a moderate effect on teacher-rated inattention problems, on internalizing problems in general, emotional reactivity symptoms, and anxious/depressed symptoms. No effect was found on intellectual abilities in either of the analyses. The authors believe these results, particularly those following ITT analyses, appear to show a slight positive effect of omega-3/omega-6 fatty acids in preschool-age children at risk of ADHD, though they alert for the need to replicate the study with larger samples to draw further conclusions. More recently, a 2022 Italian study with a total of 160 children investigated the efficacy of a specific omega-3/6 dietary supplement with omega-3/6 in ameliorating inattentive symptoms in inattentive ADHD children (6–12 years) with a baseline ADHD RS Inattention score ≥ 12 [76]. The supplement capsules contained 279 mg EPA, 87 mg DHA, and 30 mg GLA each. The study was a randomized, double-blind, placebo-controlled trial with a 6-month double-blind evaluation of omega-3/6 versus a placebo (phase 1) and a further 6-month open-label treatment with omega-3/6 on all patients (phase 2). The conclusions were that no clinical beneficial effects of omega-3/6 were detected on inattentive symptoms, suggesting a limited role of omega-3/6 dietary supplements in children with mild ADHD. 

Table 3 summarizes the results of the most recent intervention trials with PUFA supplementation in ADHD.

### 4.3. Pre-, Pro-, and Synbiotic Therapy

The development and maintenance of proper brain function is currently believed to hinge greatly on the gut microbiota, leading to the emergence of the concept of the Microbiota–Gut–Brain Axis, which illustrates how the microbiota and the brain communicate with each other [77]. Alterations in gut microbiota have increasingly been associated with psychiatric, neurologic, and neurodegenerative disorders [77,78]. In the case of ADHD, although findings are inconsistent, associations between gut microbiome features and ADHD symptoms have been reported [79], and distinct gut microbiota profiles have been found between patients with this disorder and healthy controls [79,80,81]. The variations in the findings may be attributed to differences between studies and participants in terms of age, geographical regions, and dietary patterns [79,80,81]. Indeed, considering healthy dietary patterns (rich in vegetables, fruits, fibers, and PUFAs) have been associated with a more diverse and compositionally distinct gut microbiota [82], and given the fact that dietary patterns differ geographically, gut microbiome profiles inevitably vary across populations around the world [83].

Interestingly, prebiotics and probiotics seem to offer therapeutic benefits in psychiatric disorders, including ADHD [84]. Probiotics are living bacteria strains that provide health benefits to the host, whereas prebiotics are substrates provided by the diet that are specifically metabolized by the host’s microbiota, conferring health benefits [85]. A synbiotic is a “mixture of probiotics and prebiotics that beneficially affects the host by improving the survival and implantation of live microbial dietary supplements in the gastrointestinal tract, by selectively stimulating the growth and/or by activating the metabolism of one or a limited number of health-promoting bacteria and thus improving host welfare” [86].

In a randomized double-blind, placebo-controlled prospective follow-up study, Pärtty et al. [87] found that probiotic supplementation early in life appeared to reduce the risk of ADHD and Asperger syndrome developing later in childhood. The probiotic supplementation consisted in daily *Lactobacillus rhamnosus* GG or placebo administration to the mother before expected delivery for 4 weeks and after delivery for 6 months (if the mother breastfed, otherwise the intervention was then administered directly to the child). Interestingly, the mechanisms underlying these results were not directly associated with gut microbiota composition, as there were no distinctions found between the children with these disorders and the ones without. Additionally, Kumperscak et al. [88], in a randomized double-blind, placebo-controlled prospective trial, found that probiotic supplementation with daily *Lactobacillus rhamnosus* GG/placebo administration for 12 weeks seemed to improve physical, emotional, social, and school functioning in children and adolescents with ADHD, per their own reports. Parent and teacher reports, on the other hand, suggested no improvements. More recently, in randomized double-blind, placebo-controlled trials, Sepehrmanesh et al. [89] and Ghanaatgar et al. [90] found that multi-species probiotic supplementation appeared to improve symptoms of ADHD and anxiety (but not depression), and the symptoms and severity of ADHD, respectively. This multi-species probiotic supplementation included several strains of *Lactobacillus*, *Bifidobacterium*, *Bacillus*, and *Streptococcus* (see Table 4). Additionally, in an open-label, single-arm trial, Wang et al. [91] found that probiotic supplementation with *Bifidobacterium bifidum* (Bf-688) appeared to significantly improve inattention and hyperactivity/impulsivity in children with ADHD, though a future randomized controlled trial is necessary to verify these findings.

**Table 4:** Intervention trials of probiotics and synbiotics in ADHD.

Study	Sample (N and Mean Age, in Years)	Intervention	Results
**Probiotics**			
Pärtty et al. (2015) [87]	N = 75 A = birth to 13 years old	One probiotic capsule or placebo/day 4 weeks before expected delivery 24 weeks after delivery, to the children or continuously to the mothers if breastfeeding Probiotic capsules contained 1 × 10^10^ CFU of *Lactobacillus rhamnosus* GG	At the age of 13 y.o., in the placebo group (*n* = 35), three children were diagnosed with ADHD, one with Asperger Syndrome (AS), and two with ADHD and AS. In the probiotic group (*n* = 40), 0 children were diagnosed with ADHD and/or AS.
Kumperscak et al. (2020) [88]	N = 32 A (intervention group) = 11.4 ± 3.2 A (placebo group) = 12.5 ± 2.3	One probiotic capsule or placebo/day 12 weeks Probiotic capsules contained *Lactobacillus rhamnosus* GG (at least 10^10^ CFU) and the excipients hydroxypropyl methylcellulose (E464), maltodextrins, and the coloring titanium dioxide (E171)	+ Pediatric Quality of Life Inventory (PedsQL) Child Self-Report total score x Pediatric Quality of Life Inventory (PedsQL) Parent Proxy Report total scale score x CBCL Teacher Report Form total scores
Sepehrmanesh et al. (2021) [89]	N = 34 A (intervention group) = 9.3 ± 1.3 A (placebo group) = 8.9 ± 1.0	One probiotic sachet or placebo/day 8 weeks 8 × 10^9^ CFU/day probiotic sachet with *Lactobacillus reuteri, Lactobacillus acidophilus, Lactobacillus fermentum,* and *Bifidobacterium bifidum* (each 2 × 10^9^)	+ ADHD Rating Scale (ADHD RS) + Hamilton Anxiety Rating Scale (HAM-A) x Children’s Depression Inventory (CDI)
Ghanaatgar et al. (2022) [90]	N = 38 A (intervention group) = 9.0 ± 1.8 A (placebo group) = 8.6 ± 1.7	One multi-species probiotic capsule or placebo/day 8 weeks Probiotic capsules contained 14 strains of bacteria (Bio-Kult-protexin: 2 × 10^9^ CFU/capsule), including *Bacillus subtilis* PXN 21, *Bifidobacterium bifidum* PXN 23, *Bifidobacterium breve* PXN 25, *Bifidobacterium infantis* PXN 27, *Bifidobacterium longum* PXN 30, *Lactobacillus acidophilus* PXN 35, *Lactob. delbrueckii* ssp. Bulgaricus PXN 39, *Lactob. casei* PXN 37, *Lactob. plantarum* PXN 47, *Lactob. rhamnosus* PXN 54, *Lactob. helveticus* PXN 45, *Lactob. salivarius* PXN 57, *Lactococcus lactis* ssp. *lactis* PXN 63, and *Streptococcus thermophiles* PXN 66	+ Conners Parent Rating Scale—short version (CPRS RS) scores at the 4th and 8th weeks + Clinical Global Impression-Severity scale (CGI-S) scores in all time intervals
Wang et al. (2022) [91]	N = 30 A = 6.9	One probiotic sachet in the morning and one in the evening/day 8 weeks 5 × 10^9^ CFU/day probiotic sachet with *Bifidobacterium bifidum* (Bf-688)	+ ADHD inattention and hyperactivity/impulsive symptoms assessed using the Swanson, Nolan, and Pelham Rating scale (SNAP-IV)
**Synbiotics**			
Skott et al. (2020) [92]	N = 182 n children = 68 n adults = 114 Median age children = 12 (10–14) Median age adults = 36 (29–42)	One synbiotic sachet or placebo/day 9 weeks Synbiotic 2000 (Synbiotics AB, Sweden), was a lyophilized composition of 4 × 10^11^ CFU per dose of three lactic acid bacteria: *Pediococcus pentosaceus* 5–33:3/16:1 (Strain deposit number: LMG P20608), *Lactobacillus casei ssp paracasei* F19 (LMG P-17806), *Lactobacillus plantarum* 2362 (LMG P-20606), and 2.5 g of each of the fermentable fibers beta-glucan, inulin, pectin, and resistant starch	x ADHD symptoms, daily functioning, and comorbid autism symptoms + Autism symptoms in children with vascular inflammation + Emotion regulation in adults with vascular inflammation

+ = improvements; x = no improvements.

Nevertheless, despite there being a few studies demonstrating the benefits of probiotics, particularly the *Lactobacillus rhamnosus* GG strain, in cognitive function and in health-related quality of life, there is still insufficient evidence for the recommendation of probiotic supplements as a means to treat ADHD [81]. In addition, although more recently a couple of studies demonstrated the benefits of multi-species probiotics in ADHD symptoms, the evidence is, similarly, still scarce for its recommendation. Could it be that probiotics alone are not sufficiently effective by themselves? Synbiotic supplementation could perhaps provide answers to this question. In a randomized double-blind, parallel, placebo-controlled trial, Skott et al. [92] found that synbiotic supplementation with lactic acid bacteria and fermentable fibers (beta-glucan, inulin, pectin, and resistant starch) to children and adults with ADHD for 9 weeks had no specific effect on ADHD symptoms, daily functioning, or comorbid autism symptoms. They did find, however, that synbiotic supplementation had a specific effect in those with vascular inflammation, with the children showing a reduction in autism symptoms and the adults an improvement in emotion regulation. The reduction in autism symptoms was found to be driven by the absence of ADHD medication, which the authors suggest may be due to either a milder presentation of ADHD or a possible influence of ADHD medication on the gut microbiome. 

Table 4 summarizes the results of intervention trials with probiotics and synbiotics in ADHD.

### 4.4. Specific Diets and Dietary Patterns in the Treatment of ADHD

The dietary approach to stop hypertension (DASH) is a well-known healthy eating pattern, which is characterized by high amounts of fruits, vegetables, low-fat dairy products, vitamin C, and low amounts of simple sugars. In a 12-week randomized controlled clinical trial in Iran [9], this dietary pattern appears to improve ADHD symptoms, measured by the Abbreviated Conners Scale (ACS), the 18-item Swanson, Nolan, and Pelham scale (SNAP-IV), and the Strengths and Difficulties Questionnaire (SDQ). Although there is only one study with DASH in ADHD, it is reasonable to think it could be beneficial to these patients because of its known health benefits [93,94,95,96,97,98,99].

Food elimination diets have been one of the most studied dietetic interventions in ADHD [11,12,100,101,102,103,104]. They may vary in their specific content but take three main forms: (i) a single-food exclusion diet that excludes one suspected food; (ii) a multi-food exclusion diet, such as the six-food elimination diet, which eliminates the most common food allergens; and (iii) a “few-foods diet”, such as the oligoantigenic diet, which restricts a person’s diet to only a few less commonly consumed foods, such as lamb/venison, quinoa/rice, and others with low allergenic potential. All these diets eliminate and subsequently reintroduce single foods one at a time. After the initial elimination of most food items for a limited period of time, it is expected that children show improvements in behavior or cognitive performance, and then, food items are consecutively reintroduced in a controlled way in order to determine which foods are related to adverse reactions or symptoms [100].

The elimination diet (ED) consists of a 5-week elimination period where all known food allergens—proteins from milk, egg, wheat, fish, soy, peanuts, and nuts—and potential food triggers (gluten and histamine-releasing or histamine-containing products) are eliminated. In addition, sugar intake is restricted in this period. After this phase, there is a re-introduction period that may last up to 12 months, with the objective of introducing a new food according to a standardized scheme in a sufficient amount to be able to trigger ADHD symptoms. This consists of four phases. In phase 1, food allergens are reintroduced one by one. If the reintroduction of a food allergen does not trigger recurrence of any symptoms, based on the daily assessments of parents, this food allergen is added to the diet and can be eaten again. If a food allergen does seem to trigger recurrence of ADHD symptoms, the food allergen is listed in the category ”to be avoided”. In the next week, no new food allergen introduction takes place to allow the ADHD symptoms to decrease again to the level prior to reintroduction. When ADHD symptoms have stabilized, another new food allergen is introduced in the week thereafter. In the following phases, sugar (phase 2), histamine-releasing or histamine-containing products (phase 3), and additives (phase 4) are reintroduced. The procedures during reintroduction phases are all similar to phase 1 [101].

There are two recent studies with an elimination diet in children with ADHD. The TRACE study [101] is an ongoing two-arm RCT comparing the short- and long-term effects of an elimination diet and a healthy diet in children with ADHD, which is occurring in the Netherlands, and may bring new insights into the long-term effects of dietary treatments for ADHD. The INCA study [104] is a RCT that consisted of an open-label phase with masked measurements followed by a double-blind crossover phase, where children aged 4–8 years who were diagnosed with ADHD were randomly assigned to 5 weeks of a restricted elimination diet (diet group) or a healthy diet (control group). Children in the diet group who had behavioral improvement of at least 40% on the parent ADHD Rating Scale (ARS)—the clinical responders—entered the challenge phase; the non-responders left the trial. Based on the levels of IgG (μg/mL) in serum, each analyzed food was categorized as a low-IgG food or a high-IgG food. The diet group responders, in the second phase (double-blind crossover challenge phase; weeks 10 to 13), re-introduced two groups of foods consisting of either three high-IgG or three low-IgG foods, each for 2 weeks. These foods added in the challenge phase were individually chosen and differed per child. All behavioral measurements in the challenge phase were double-blind and the ARS and the Abbreviated Conners Scale (ACS) assessments were done after each challenge. The total ARS score increased in clinical responders after the challenge by 20.8 (95% CI 14.3–27.3; *p* < 0.0001) and the ACS score increased by 11.6 (7.7–15.4; *p* < 0.0001). After challenges with either high-IgG or low-IgG foods, relapse of ADHD symptoms occurred in 63% of children, independent of the IgG blood levels. This study shows that a strictly supervised restricted elimination diet could be a valuable instrument to assess whether ADHD behaviors are induced by food, but the prescription of diets using IgG blood tests should be discouraged.

Pelsser et al. [103] analyzed unpublished data from the INCA study and the *Biomarker Research in ADHD: the Impact of Nutrition* [BRAIN] study and found an association between the Few-Foods Diet (FFD) and a decrease in thermoregulation problems, gastrointestinal complaints, eczema, and sleep problems. The INCA results show a clinically relevant reduction in the FFD group compared to the control group and the open-label BRAIN results confirmed the outcomes of the FFD group. However, no association was detected between the decrease in physical complaints and the decrease in ADHD symptoms. In a systematic review of meta-analyses of double-blind, placebo-controlled trials, Pelsser et al. [12] also found that the effect size of an FFD in parent ratings was 0.80 (95% CI: 0.41–1.19, I2 = 61%) and 0.51 (95% CI: −0.02–1.04, I2 = 72%) in others ratings, showing that this diet could potentially offer a treatment opportunity in subgroups of children with ADHD not responding to or too young for medication.

The oligoantigenic diet (OD) is another restrictive diet that consists in the elimination of individually allergenic food items from the diet. Dölp et al. [102] found that this diet improves children’s ADHD Rating Scale (ARS) scores, measured by video-rating. Participants with >40% improvement in the ARS between T1 (before the diet) and T2 (after the diet) were defined as responders. Nutrients with individual relevance to ADHD symptoms were identified in a following reintroduction phase (T3–T4) lasting 8–16 weeks. The ARS was completed by a non-blinded child and adolescent psychiatrist. Then, reintroduction sessions were recorded on video, pseudonymized, and evaluated by three raters (two blinded raters and one non-blinded). Complete data were captured for eight children. The three raters considered four of the eight children as responders to the OD; however, RCTs are necessary to assess this association.

## 5. Discussion

The findings of the observational studies emphasize a potential role of dietary patterns in ADHD; however, these study designs are unable to establish a causal relationship between diet and ADHD. Moreover, associations between adherence to healthy diets and low prevalence of ADHD do not necessarily imply healthy foods consumed during childhood have a protective effect. The associations between dietary habits and ADHD risk may be caused by other factors that were not recorded. Lifestyle factors (e.g., physical activity, screen time, and sleep) influence dietary patterns and may be important factors in ADHD symptomatology [105]. Even when statistical adjustment for potential confounding variables was performed, residual confounding was still unavoidable. In addition, the commonly used food frequency questionnaires are known to contain some degree of measurement error [106]. In many of the included studies in this review, the dietary patterns derived from principal component analysis explained less than 50% of total variance, suggesting the influence of other patterns and factors.

While some studies reported that dietary supplements have beneficial effects, severe methodological limitations were observed, such as extremely short intervention periods, as well as a lack of randomization, placebo, and prospective monitorization of the long-term effects of the supplementation. Increasing evidence suggests that treating ADHD with diet interventions might be particularly useful for specific subgroups of children and adolescents, but more studies about the effects of these diet interventions in ADHD are still needed.

From the clinical point of view, restriction and elimination diets can lead to nutritional deficiencies and, consequently, poor growth in children with ADHD, so the nutritional status of these children must be rigorously monitored [100], and these diets must be used with caution. These diets can be compared to the low-FODMAP diet used in Irritable Bowel Syndrome [107,108,109,110], which is also a highly restrictive diet that has proven efficacy but is only used in specific cases due to its high risk of causing nutritional deficiencies.

To the best of our knowledge, elimination diets as part of ADHD treatment are not currently used in clinical practice; however, they could potentially be useful in specific cases of children resistant to the medication or too young to start it. Nevertheless, these interventions, as for all dietary interventions, do not work for all patients, so a precision/personalized medicine approach must be considered [11], especially in ADHD due to its phenotypic heterogeneity [1]. Future studies should attempt to identify subgroups of individuals diagnosed with ADHD who may benefit from these diets.

## 6. Strengths and Limitations

In this narrative review, we aimed to summarize the existing evidence for the clinical use of dietary interventions in ADHD patients and review which dietary patterns are most associated with ADHD. As such, we selected and described the most recent high-quality studies within this line of research in order to examine this topic. Nonetheless, as this is a narrative review, we merely provided a description and critical reflection of the current knowledge. Hence, unlike in a systematic review, a detailed search strategy along with an objective assessment of the quality of the studies and the robustness of their results was not carried out. Still, we predominantly reported data from RCTs, systematic reviews, and meta-analyses, which have the highest level of evidence.

## 7. Conclusions

In ADHD, dietary patterns appear to play a significant role in the risk of developing or aggravating disease symptoms, with unhealthy patterns being most positively associated with this pathology, and healthy eating patterns being inversely associated with ADHD. Altered levels of nutrients, such as vitamin D, iron, zinc, and PUFAs, have also been associated with the aggravation and progression of ADHD. Therefore, diet has emerged as a treatment option for ADHD. Notwithstanding, more robust scientific evidence is required for these dietary interventions to be implemented as part of ADHD therapy.

## Figures and Tables

**Table 1:** Intervention trials of nutritional supplements in ADHD.

Study	Sample (N and Mean Age, in Years)	Intervention	Results
**Zinc**			
Noorazar et al. (2020) [63]	N = 60 A = 9.67	10 mg zinc/day 6 weeks	+ Conners Inattention score
Zamora et al. (2011) [64]	N = 40 A = 9.8	10 mg zinc/day 6 weeks	+ Conners score—teacher version
Arnold et al. (2011) [65]	N = 52 A = 9.8	15 mg zinc/day 8 weeks double-blind zinc/placebo (phase 1) 2 weeks open-label fixed-dose amphetamine (phase 2) 3 weeks double-blind continuation + amphetamine titration to optimal dose (phase 3)	Clinical outcomes were equivocal, sometimes favoring zinc, sometimes the placebo, but objective neuropsychological measures mostly favored b.i.d. zinc
Akhondzadeh et al. (2004) [66]	N = 44 A = 7.9	15 mg zinc/day 6 weeks	+ ADHD-RS Parent and Teacher scores
Bilici et al. (2004) [67]	N = 400 A = 9.6	40 mg zinc/day 12 weeks	+ Hyperactive, impulsive, and socialization symptoms
**Iron**			
Konofal et al. (2008) [68]	N = 22 A = 5.9	80 mg/day ferrous sulfate 12 weeks	+ ADHD Rating Scale (ADHD-RS) and Clinical Global Impression-Severity (CGI-S) scores x Conners tests
Panahandeh et al. (2017) [69]	N = 42 A = 8.9	5 mg/kg/day (ferrous sulfate) 8 weeks	+ CSI-4 total and factor scores
Rucklidge et al. (2018) [70]	N = 93 A = 9.7	Zinc (3.2 mg/capsule) Iron (0.9 mg/capsule) Dose: starting with 3 and increasing to 12 capsules/day 10 weeks	+ Inattentive levels x Hyperactive-impulsive symptoms
**Vitamin D**			
Hemamy et al. (2021) [60]	N = 66 A = 9.1	50,000 IU/week 25-hydroxy-vitamin D3 + 6 mg/kg/day magnesium 8 weeks	+ Emotional problems, conduct problems, peer problems, prosocial score, total difficulties, externalizing and internalizing scores
Dehbokri et al. (2019) [59]	N = 96 A = 9.2	50,000 IU/week 25-hydroxy-vitamin D3 6 weeks	+ Conners and all subscale scores, x for children with a sufficient baseline level of vitamin D
Mohammadpour et al. (2018) [71]	N = 54 A (intervention group) = 7.70 ± 1.77 A (placebo group) = 8.03 ± 1.44	2000 IU/day 25-hydroxy-vitamin D3 8 weeks	+ Evening symptoms and total score of Weekly Parent Ratings of Evening and Morning Behavior scale x Conners Parent Rating Scale Revised and ADHD Rating Scale-IV scores
Elshorbagy et al. (2018) [72]	N = 35 A (intervention group) = 9.31 ± 2.60 A (placebo group) = 8.80 ± 3.72	3000 IU/day 25-hydroxy-vitamin D3 12 weeks	+ Cognitive function at the conceptual level, inattention, opposition, hyperactivity, and impulsivity domains
Naeini et al. (2019) [73]	N = 71 A (intervention group) = 9.20 ± 1.84 A (placebo group) = 9.04 ± 1.2	1000 IU/day 25-hydroxy-vitamin D3 12 weeks	+ Conners Parent Questionnaire (CPQ), Strengths and Difficulties Questionnaire (SDQ) completed by parents, and SDQ completed by teachers + Impulsivity mean scores of the Continuous Performance Test (CPT) x Attention and mean reaction time mean scores of the CPT

+ = improvements; x = no improvements.

**Table 2:** Meta-analyses of intervention trials with PUFAs in ADHD.

Authors	Studies	Results
Chang et al. (2018) [57]	Seven RCTs assessing the effects of *n*-3 PUFAs in clinical symptoms *n* = 534	+ ADHD clinical symptoms scores reported by parents x ADHD severity reported by teachers
	Three RCTs assessing the effects of *n*-3 PUFAs in cognitive performance *n* = 214	+ Cognitive measures associated with attention (omission and commission errors) x Memory and information processing
Händel et al. (2021) [74]	31 RCTs assessing PUFA supplementation N = 1775	x ADHD core symptoms rated by parents or teachers x Behavioral difficulties rated by parents or teachers x Quality of life

+ = improvements; x = no improvements.

**Table 3:** Intervention trials of PUFAs in ADHD.

Study	Sample (N and Mean Age, in Years)	Intervention	Results
Döpfner et al. (2021) [75]	N = 40 A (intervention group) = 5.5 ± 0.61 A (placebo group) = 4.97 ± 0.93	Two capsules of an Omega-3/Omega-6 fatty acid supplement twice a day, corresponding to a daily dose of 372 mg EPA, 116 mg DHA, and 40 mg GLA 16 weeks	+ Parent- and teacher-rated ADHD symptoms, parent-rated internalizing symptoms, and parent- and teacher-rated externalizing symptoms in intention-to-treat analyses + Teacher-rated inattention symptoms and parent-rated internalizing problems in analyses involving all available data x Intellectual abilities in either of the analyses
Carucci et al. (2022) [76]	N = 160 Age = 9.7 ± 1.9	Two capsules containing 279 mg EPA, 87 mg DHA, 30 mg GLA (gamma linolenic acid) or placebo/day 6 months double-blind (phase 1) 6 months open-label (phase 2)	x ADHD RS inattention score after phase 1 + ADHD RS total score after 12 months

+ = improvements; x = no improvements.

## Data Availability

Not applicable.
